# Antimicrobial Resistance in *Lactococcus* spp. Isolated from Native Brazilian Fish Species: A Growing Challenge for Aquaculture

**DOI:** 10.3390/microorganisms12112327

**Published:** 2024-11-15

**Authors:** Angélica Emanuely Costa do Rosário, Angelo Carlo Chaparro Barbanti, Helena Caldeira Matos, Cynthia Rafaela Monteiro da Silva Maia, Júlia Miranda Trindade, Luiz Fagner Ferreira Nogueira, Fabiana Pilarski, Silvia Umeda Gallani, Carlos Augusto Gomes Leal, Henrique César Pereira Figueiredo, Guilherme Campos Tavares

**Affiliations:** 1 Post-Graduate Program in Aquaculture, Nilton Lins University, Manaus 69058-030, AM, Brazil; angelicamanu0807@gmail.com (A.E.C.d.R.); angelocarloch@gmail.com (A.C.C.B.); cynthiarafaeladasilva.2023@gmail.com (C.R.M.d.S.M.); silviaugallani@gmail.com (S.U.G.); 2 Department of Preventive Veterinary Medicine, School of Veterinary Medicine, Federal University of Minas Gerais—UFMG, Belo Horizonte 31270-901, MG, Brazil; caldeiramatosh@gmail.com (H.C.M.); juliamirandatrindade@outlook.com (J.M.T.); fagnerfnogueira@outlook.com (L.F.F.N.); leal.cag@gmail.com (C.A.G.L.); figueiredoh@yahoo.com (H.C.P.F.); 3 Laboratory of Microbiology and Parasitology of Aquatic Organisms, Aquaculture Center of Unesp, São Paulo State University (Unesp), Jaboticabal 14884-900, SP, Brazil; fabiana.pilarski@unesp.br

**Keywords:** disk diffusion, epidemiological cutoff values, fish, piscine lactococcosis

## Abstract

*Lactococcus* spp. has emerged as a pathogen that is affecting global aquaculture, with *L. garvieae*, *L. petauri*, and *L. formosensis* causing piscine lactococcosis. While antimicrobials are commonly used to treat diseases in aquaculture, reports of antimicrobial resistance in fish isolates are increasing. However, little is known about the susceptibility patterns of *Lactococcus* spp. strains isolated from native fish species in Brazil. This study aimed to assess the antimicrobial susceptibility of these strains and establish a provisional epidemiological cutoff value for *L. garvieae* using the normalized resistance interpretation approach. A total of 47 isolates were tested: 17 *L. garvieae*, 24 *L. petauri*, and 6 *L. formosensis*. The isolates were classified as wild-type (WT) or non-wild-type (NWT) based on inhibition zone diameters. Isolates classified as NWT for three or more antimicrobial classes were considered multidrug-resistant, and the multiple antibiotic resistance (MAR) index was calculated. The results revealed heterogeneity in antimicrobial resistance profiles, with higher resistance to trimethoprim/sulfamethoxazole and norfloxacin. Resistance to other antimicrobials, including florfenicol and oxytetracycline (approved for use in Brazil), varied according to the bacterial species. *Lactococcus petauri* (87.5%) and *L. formosensis* (66.7%) showed the highest multidrug resistance, compared to *L. garvieae* (11.7%), along with higher MAR index values. These findings suggest that multidrug-resistant strains could pose future challenges in the production of native species, underscoring the need for ongoing monitoring of antimicrobial resistance and responsible use of antimicrobials in aquaculture.

## 1. Introduction

Piscine lactococcosis is considered an emerging bacterial disease for fish farming worldwide [1], and the number of hosts in which *Lactococcus garvieae*, *L. petauri*, and *L. formosensis* has been detected has expanded [2,3,4,5,6,7]. The disease is currently a significant health challenge for *Oncorhynchus mykiss* (*O. mykiss*) and *Oreochromis niloticus* (*O. niloticus*) production, causing high mortality rates and significant economic losses [8,9,10].

One of the main methods for controlling outbreaks of bacterial diseases in fish farms is antibiotic therapy [11]. Different classes of antimicrobials have been commonly used in global aquaculture, such as tetracyclines, phenicols, quinolones, β-lactams, macrolides, aminoglycosides, and sulfonamides. Each country has its own legislation governing the approval of antimicrobials for specific animal species, as well as regulations on the usage practices and allowable residue limits in animal-derived products [12]. However, none of these drug classes were developed exclusively for use in aquatic animals, and only a few antimicrobials are approved for use in this animal production sector [11]. For example, in Brazil, only oxytetracycline and florfenicol are permitted for use in aquaculture [13]. However, there are already reports of *Lactococcus* spp. strains becoming resistant to the main drugs used in aquaculture [14,15]. The indiscriminate use of antimicrobials has been reported by producers and technicians from different fish farms, which can result in bacterial resistance to specific drugs [16]. As a result, a product already used by a producer may no longer be effective in treating bacteriosis, thereby necessitating the use of another antibiotic. Additionally, it is worth mentioning that the rate of approval for new drugs is slower than the evolution of bacterial resistance, leading producers to use off-label drugs [17].

One way to monitor antimicrobial susceptibility in lactococcosis-causing bacteria is through the use of laboratory methods, such as disk diffusion [10] and broth dilution [18] methods. The former is considered an inexpensive, reliable, and simple technique that can be easily applied in a laboratory routine, while in comparison, the latter is technically demanding and labor-intensive [19]. However, in Brazil, few studies have evaluated these methodologies for testing *Lactococcus* spp. strains, whether using isolates from terrestrial mammals or aquatic animals. In Brazil, the disk diffusion assay has been performed for isolates of *L. petauri* from farmed *O. niloticus*, and resistance for some isolates to amoxicillin, erythromycin, florfenicol, and norfloxacin was identified. In addition, all the isolates evaluated were considered resistant to trimethoprim/sulfamethoxazole [10]. Bacteria of the genus *Lactococcus* have also been isolated from native Brazilian fish species [2], and little is known about the use of antimicrobials in these species and their antimicrobial susceptibility profiles.

The main problem in determining the sensitivity of bacterial fish pathogens to antimicrobials is the lack of reference values. Without these values, it is not possible to determine whether an isolate is sensitive or resistant. There are no internationally recognized epidemiological cutoff values for disk diffusion data for *Lactococcus* spp. strains in the Clinical Laboratory Standards Institute (CLSI) or the European Committee on Antimicrobial Susceptibility Testing (EUCAST) guidelines. Previous studies have generated provisional epidemiological cutoff values for *L. petauri* from disk diffusion zone data using the normalized resistance interpretation (NRI) method [10] for *L. garvieae* and *L. petauri* from minimum inhibitory concentration data using NRI and ECOFFinder approaches [18]. Nevertheless, for disk diffusion zone data, there are no reports of established cutoff values in the literature for *L. garvieae* and *L. formosensis*.

Therefore, the aim of this study was to evaluate the susceptibility profile of *L. formosensis*, *L. garvieae*, and *L. petauri* strains obtained from native Brazilian fish species to different antimicrobials and to calculate the provisional epidemiological cutoff values (pECVs) for *L. garvieae*.

## 2. Materials and Methods

### 2.1. Bacterial Strains and Identification

A total of 47 *Lactococcus* spp. strains (*n* = 6 *L. formosensis*, *n* = 17 *L. garvieae*, and *n* = 24 *L. petauri*) were used in this study. The isolates were obtained from 11 native fish species (*Arapaima gigas*, *Brycon amazonicus*, *Cichla* sp., *Colossoma macropomum*, *Hoplias macrophtalmus*, *Hoplias malabaricus*, *Lophiosilurus alexandri*, *Phractocephalus hemioliopterus*, *Pseudoplatystoma corruscans*, *Pseudoplatystoma fasciatum*, and a hybrid of *Pseudoplatystoma*) originating from free-living fish or commercial farms, between 2012 and 2024, in 6 Brazilian states (Amazonas, Bahia, Mato Grosso do Sul, Minas Gerais, Pará, and São Paulo) (Table 1) [2,20,21,22,23,24]. These isolates were obtained through routine laboratory diagnosis of bacterial diseases in fish conducted by the Laboratory of Aquatic Animal Diseases (Veterinary School, Federal University of Minas Gerais, Belo Horizonte, Brazil), Laboratory of Applied Microbiology of Aquatic Organisms (Nilton Lins University, Manaus, Brazil), Laboratory of Microbiology and Parasitology of Aquatic Organisms (Aquaculture Center of São Paulo State University, São Paulo, Brazil), and Fisheries Institute (São Paulo, Brazil). Of these, 11, originating from *Arapaima gigas* (*n* = 7), *Cichla* sp. (*n* = 1), *Hoplias malabaricus* (*n* = 1), and *Pseudoplatystoma* sp. (*n* = 2), were recovered through bacterial examination after the recent disease outbreak. Furthermore, all the selected isolates were identified to species level using matrix-assisted laser desorption ionization time-of-flight (MALDI-TOF) mass spectrometry (Bruker Daltonics, Bremen, Germany) [22] with the Bruker MALDI Biotyper database (v13.0.0.2), followed by *gyrB* sequencing [25]. The isolates were stored at −80 °C in BHI broth with 15% glycerol until use.

### 2.2. Susceptibility Testing

Disk diffusion tests against *Lactococcus* spp. were carried out according to the protocol provided in the CLSI guideline VET03, with adaptations recommended for bacteria of the genus *Streptococcus* (Group 4) [26]. The disks used contained 10 µg amoxicillin, 15 µg erythromycin, 30 µg florfenicol, 10 µg neomycin, 10 µg norfloxacin, 30 µg oxytetracycline, and 1.25/23.75 µg trimethoprim/sulfamethoxazole. The disks were obtained from a commercial company (Oxoid, Hampshire, UK).

The selected isolates (Table 1) were thawed, inoculated onto Man Rogosa & Sharpe (MRS, Merck, Darmstadt, Germany) agar, and incubated at 28 °C for 48 h. After incubation, colonies were collected and suspended in a sterile saline solution until they reached an absorbance of between 0.08 and 0.13 (DO_625_) nm using a spectrophotometer (Spectrum, Shanghai, China). Muller–Hinton agar enriched with 5% defibrinated sheep blood was inoculated with the bacterial suspension using sterile swabs. Then, antimicrobial disks were placed on the agar, and the plates were incubated at 28 °C for 24 h. Additionally, the quality control reference strains *Escherichia coli* ATCC 25922 and *Aeromonas salmonicida* subsp. *salmonicida* ATCC 33658 were grown on blood agar, incubated at 28 °C for 24 h, and subjected to the same experimental conditions described above as recommended by the CLSI for this method. All the procedures were performed in duplicate. The diameter of the inhibition zone of all the isolates was measured.

### 2.3. Calculation of Provisional Lactococcus Garvieae Epidemiological Cutoff Values

As there is no established zone diameter cutoff for *L. garvieae* generated by a standard method, this study calculated the pECV for each antimicrobial agent tested using the automatic normalized resistance interpretation (NRI) method (www.bioscand.se/nri (accessed on 17 August 2024)) from the inhibition zone data that was generated [27,28]. The isolates were then classified as wild-type (WT) or non-wild-type (NWT) [29]. To meet the minimum requirements of the NRI method [30], the disk diffusion data of the *L. garvieae* strains isolated from *O. niloticus* (*n* = 3), *Trichogaster lalius* (*n* = 1), and *Xiphophorus maculatus* (*n* = 1) from the Laboratory of Aquatic Animal Diseases culture collection were included in the calculation of the pECVs (Table S2).

### 2.4. Data Analysis

*Lactococcus petauri* strains were classified as WT or NWT according to the previously established pECV [10]. However, as *L. formosensis* has no number of suggested observations to set a reliable pECV calculation, the inhibition zone data were shown as maximum, minimum, mean, and standard deviation values. Regardless of the bacterial species, bacteria that did not present an inhibition zone and did not have a defined ECV were considered not susceptible (NS) for the antimicrobials [31]. R software v.4.3.1 [32] and RAWGraphs v2.0 [33] were used for data visualization. Isolates classified as NWT for at least three classes of antimicrobials were considered to be multidrug-resistant bacteria [34]. The multiple antibiotic resistance (MAR) index was also calculated [35].

## 3. Results

### 3.1. Bacterial Identification

From the sequencing of the *gyrB* gene, *Lactococcus formosensis* (*n* = 1, *Arapaima gigas*), *L. garvieae* (*n* = 1, *Cichla* sp.; *n* = 1, *Hoplias malabaricus*; *n* = 1, *Pseudoplatystoma* sp.), and *L. petauri* (*n* = 6, *Arapaima gigas*; *n* = 1, *Pseudoplatystoma* sp.) were detected from the current disease outbreaks in native fish species (Table 1). The *gyrB* gene sequences of these isolates were included in the NCBI database.

The remaining isolates used in this study were identified at the species level in a previous study [2].

### 3.2. Quality Control

The reference strains *E. coli* ATCC 25922 and *Aeromonas salmonicida* subsp. *salmonicida* ATCC 33658 presented inhibition zone diameters within the acceptable ranges established by the CLSI (Table 2).

### 3.3. Antimicrobial Susceptibility for Lactococcus formosensis

**Figure 1:**
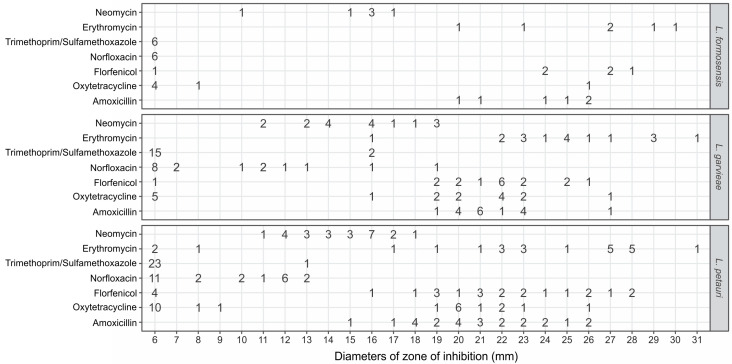
Disk diffusion scatter plots for antimicrobials versus diameters of inhibition zones for the six *L. formosensis*, 17 *L. garvieae*, and 24 *L. petauri* strains evaluated.

**Figure 2:**
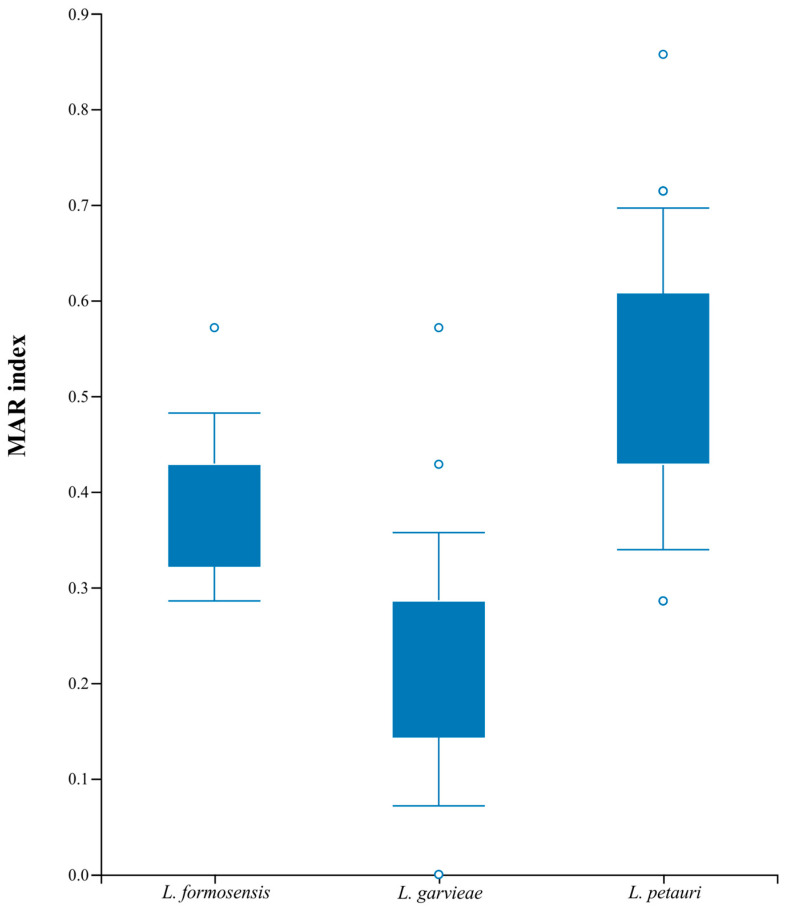
Multiple antibiotic resistance (MAR) index box plot of *Lactococcus* spp. strains isolated from native Brazilian fish species.

The disk diffusion assay for *L. formosensis* strains exhibited zones ranging between 6 mm and 30 mm (Table 2 and Table S1). All the isolates were categorized as NS (no observation of inhibition zone) for trimethoprim/sulfamethoxazole and norfloxacin (Figure 1). A total of one and four strains were categorized as NS for florfenicol and oxytetracycline, respectively (Figure 1). For these antimicrobials, all the isolates from *A. gigas* were categorized as NS for oxytetracycline, and the LG91-23 strain (from *Pseudoplatystoma* sp.) was NS for both drugs (Figure S1). Since there is no pECV for *L. formosensis*, it is not possible to determine whether the other isolates are resistant to other antimicrobials. In addition, four isolates were classified as multidrug-resistant. The MAR index of the isolates varied between 0.285 and 0.571 (Figure 2, Table S1).

### 3.4. Antimicrobial Susceptibility for Lactococcus garvieae

The disk diffusion assay for *L. garvieae* strains exhibited zones ranging between 6 mm and 31 mm (Table 2 and Table S1). The distribution of the inhibition zones obtained from all the evaluated isolates are shown in Figure S2. The calculated pECVs for the antimicrobials are presented in Table 2. A total of 8 and 15 isolates (Figure 1) presented a zone of complete inhibition of 6 mm for norfloxacin and trimethoprim/sulfametoxazole, respectively, preventing the establishment of the ECV for these antimicrobials. However, these isolates were categorized as NS. Based on the calculated pECVs, all the isolates were classified as WT for amoxicillin, erythromycin, and neomycin. One and five isolates were classified as NWT for florfenicol and oxytetracycline, respectively, especially those strains isolated from *Pseudoplatystoma* sp. (Figure S1). In addition, two isolates were classified as multidrug-resistant. The MAR index of the isolates varied between 0.00 and 0.428 (Figure 2, Table S1).

### 3.5. Antimicrobial Susceptibility for Lactococcus petauri

The disk diffusion assay for the *L. petauri* strains exhibited zones ranging between 6 mm and 31 mm (Table 2 and Table S1). All the isolates were classified as WT for neomycin. A total of 1, 8, 11, 22, 22, and 23 isolates were classified as NWT for amoxicillin, erythromycin, florfenicol, oxytetracycline, norfloxacin, and trimethoprim/sulfametoxazole, respectively (Table S1). A resistance phenotype for florfenicol and oxytetracycline was observed in all the native Brazilian fish species in which *L. petauri* was isolated (Figure S1). A total of 21 isolates were classified as multidrug-resistant. The MAR index varied between 0.285 and 0.857 (Figure 2, Table S1).

## 4. Discussion

Currently, antimicrobial resistance is one of the biggest threats to public health [36], especially with the emergence of multidrug-resistant strains [37]. The antimicrobial susceptibility profile in lactococcosis-causing bacteria strains has been studied by several different institutions and researchers using various techniques, such as disk diffusion [38,39], broth dilution [14,40], and the Etest [41]. Studies have suggested that most of the isolates evaluated are resistant to ampicillin [39,42], florfenicol [43], flumequine [38], nalidixic acid [42,44,45], norfloxacin [43], tetracycline [15], and trimethoprim/sulfamethoxazole [38,42,44,46]. Although detected at lower percentages, there are also records of resistance to amoxicillin (16–23%), bacitracin (42%), ciprofloxacin (4%), chloramphenicol (18%), enrofloxacin (33–67%), erythromycin (16–52%), kanamycin (33%), oxytetracycline (4–44%), and streptomycin (33%) [15,38,39,42,45,46].

In the scientific literature, it is possible to observe heterogeneity in the antimicrobial resistance profiles for *Lactococcus* spp. strains, which may be related to the different species within the genus. This is because most of the articles, including some recent ones, did not perform the correct taxonomic classification of the isolates, which is currently recommended [47]. Only three studies assessed the antimicrobial resistance profile after correct species identification, using disk diffusion [10,48] and broth dilution [18,49]. Furthermore, Öztürk et al. [18] suggest that this heterogeneity is related to the overuse or misuse of antimicrobials at the farm level and the lack of established susceptibility cutoff values for each of the three species that cause piscine lactococcosis. Here, we evaluated the antimicrobial resistance profiles of *L. formosensis*, *L. garvieae*, and *L. petauri* strains isolated from native fish species in Brazil using disk diffusion susceptibility testing and established pECVs for five out of seven antimicrobials for *L. garvieae* strains.

Regardless of the bacterial species evaluated in our study, the trimethoprim/sulfametoxazole resistance phenotype stood out (*L. formosensis* = 100%, *L. garvieae* = 88.2%, and *L. petauri* = 95.8%). Resistance to this drug has previously been reported in the literature for *Lactococcus* spp. strains isolated from *O. mykiss*, *Dicentrarchus labrax*, and *O. niloticus* [6,10,38,42,43,44,46]. For the other drugs, interspecies variation has been observed.

Unfortunately, due to the limited number of isolates identified as *L. formosensis* in our study, it was not possible to establish a pECV; therefore, classification as WT or NWT could not be performed. However, we considered those isolates for which no inhibition zones were observed for the antimicrobials tested to be NS. Thus, in addition to trimethoprim/sulfametoxazole, all the isolates were considered NS for norfloxacin. This result is in disagreement with the study conducted by Lin et al. [50], in which all the *L. formosensis* strains isolated from milk samples of a cow with clinical mastitis were susceptible to quinolones via broth dilution testing. We also did not observe the formation of inhibition zones in four isolates (three from *A. gigas* and one from *Pseudoplatystoma* sp.) for oxytetracycline, nor in one *Pseudoplatystoma* sp. isolate (LG91-23) for florfenicol. Chan et al. [48] evaluated susceptibility using the disk diffusion method for an *L. formosensis* strain obtained from a human with bacteremia and found that the isolate was susceptible to tetracycline. There is no mention in the literature regarding resistance profiles to florfenicol, regardless of the host evaluated; however, a previous study demonstrated resistance to another amphenicol, chloramphenicol, for all the isolates evaluated [50]. Although we cannot determine susceptibility for other drug classes, the literature mentions *L. formosensis* resistance to aminoglycosides and macrolides and susceptibility to β-lactams [48,50]. If we consider the pECV of *L. garvieae* and *L. petauri* from this study, all the isolates would be classified as WT for amoxicillin, erythromycin, florfenicol, and neomycin, which would corroborate the previous information. Additionally, the AM-LG05 strain would be classified as NWT for oxytetracycline, increasing the number of multidrug-resistant isolates. It was possible to observe that the antimicrobial resistance profile was similar among the *A. gigas* isolates, as all the isolates share the same geographic origin.

For the *L. petauri* strains, we compared the results using the previously established pECV. All the isolates evaluated were classified as WT for neomycin, thereby corroborating with Egger et al. [10]. For amoxicillin and erythromycin, our isolates demonstrated a low frequency of NWT detection, 4.1% and 33.3%, respectively, when compared to other antimicrobials. A previous study demonstrated resistance of 6% and 25% for *L. petauri* strains isolated from *O. mykiss* for erythromycin and amoxicillin, respectively [18]. For isolates obtained from *O. niloticus*, the NWT percentages were lower, around 3% for both antimicrobials [10]. A high percentage of isolates classified as NWT for norfloxacin was observed in our study (91.7%) compared to the results obtained in *O. niloticus* (16.75%) [10]. Regarding florfenicol and oxytetracycline, 11.4% and 91.6% of the isolates were classified as NWT, respectively. In *O. mykiss* and *O. niloticus* isolates, NWT values of 0% and 12.5% for oxytetracycline and 3.4% to 9.4% for florfenicol have been observed [10,18]. The resistance profile was similar among the *Pseudoplatystoma* sp. isolates from the states of Mato Grosso and Minas Gerais, as well as most of the *A. gigas* isolates from Bahia. However, for isolates obtained from this latter fish species in the northern region of Brazil, individual variation was detected, as were the cases with *C. macropomum* and *B. amazonicus* isolates.

There are no studies that have standardized cutoff values, even provisional ones, for *L. garvieae* strains following its correct taxonomic identification. Therefore, our study is the first to do so. However, we emphasize that the ECVs presented here are provisional. To generate ECVs that are relevant to disk diffusion data for *L. garvieae*, a larger number of isolates (over 100 observations) from at least 5 different laboratories would be required [30]. As previously mentioned, all the isolates evaluated were classified as being WT for amoxicillin, erythromycin, and neomycin. In contrast, isolates from *O. mykiss* exhibited varying susceptibility to these antimicrobials. Approximately 2.6%, 24.4%, and 6.4–11.5% of the isolates were resistant to amoxicillin, erythromycin, and aminoglycosides, respectively [18]. A total of 47% of the isolates from native fish species in Brazil were considered to be NS for norfloxacin, in contrast to previous studies that reported low (7.7%) or no resistance to quinolones [18,48]. A total of 5.8% and 29.4% of the isolates were classified as NWT for florfenicol and oxytetracycline, respectively. However, the literature reports resistance rates of 26.9% for florfenicol and 17.9% for oxytetracycline [18]. The antimicrobial resistance profile observed in our study for the *L. garvieae* strains was not consistent among the aquatic host species analyzed or with the origin of the isolates, demonstrating a heterogeneous profile.

Our study showed that the *L. garvieae* strains tend to be more sensitive to antimicrobials when compared to the *L. formosensis* and *L. petauri* strains. Furthermore, the proportion of *L. garvieae* isolates with a MAR index greater than 0.3 (11.7%) was lower than that found in *L. formosensis* (66.7%) and *L. petauri* (87.5%). The most efficient measure to control bacterial diseases is the use of antimicrobials [11]. However, since the isolates evaluated in this study were classified as multi-resistant to several antimicrobials, treating piscine lactococcosis in native Brazilian fish species becomes challenging. Unfortunately, little is known about the use of antimicrobials in native fish species in Brazil. However, prophylactic and metaphylactic use of antimicrobials, especially oxytetracycline, in larviculture of native species and during the feeding training of carnivorous species like *Pseudoplatystoma* sp. and *A. gigas* has been reported by producers and technicians in the country. Additionally, it is known that commercial fish farming in Brazil involves off-label use of amoxicillin, enrofloxacin, and norfloxacin [17,51]. In the central-western region of Brazil, fluoroquinolones, especially norfloxacin and enrofloxacin, intended for the treatment of cattle, have also been used off-label in *Pseudoplatystoma* sp. Therefore, the widespread use of these antimicrobials may have contributed to the increase in resistance among *Lactococcus* spp. strains. It is also worth mentioning that when MAR index values exceed 0.2 (in our case, over 0.3 due to the number of antibiotics tested), a high environmental risk of spreading antimicrobial resistance is predicted [35]. In this context, the shared production of native fish species and *O. niloticus* could pose a risk of transmitting antimicrobial-resistant *Lactococcus* spp. strains, or it could enable *L. petauri* isolates from *O. niloticus* to acquire resistance genes in this production environment, resulting in an unsatisfactory therapeutic approach during disease outbreaks. Therefore, monitoring of antimicrobial resistance in *Lactococcus* spp. strains becomes essential.

Sun et al. [52] reported that acquired resistance in microorganisms occurs for two reasons: the natural resistance of bacteria to certain antimicrobials and acquired resistance due to continuous exposure to antimicrobials. Once the bacteria becomes resistant, this resistance can be transferred to the other bacterial species through horizontal gene transfer [12]. Furthermore, some *L. garvieae* strains carry these antimicrobial resistance genes on transferable R plasmids [38]. The acquisition and transfer of antimicrobial resistance genes have been considered to be responsible for the spread and distribution of antimicrobial resistance [18]. Previous studies indicate a high prevalence of antimicrobial resistance genes in *Lactococcus* spp. isolates from *O. mykiss* [15,18,38]. There is no description of the detection of resistance genes in *Lactococcus* spp. strains from Brazilian fishes. However, given the higher percentage of multi-resistant isolates, future studies should be conducted to identify resistance genes, particularly those encoding antimicrobial resistance, using genomic tools.

In Brazil, only florfenicol and oxytetracycline are approved antimicrobial agents for use in aquaculture [13]. Both antimicrobials act against Gram-negative and Gram-positive bacteria; they are bacteriostatic drugs that work by binding to bacterial ribosomal subunits and inhibiting protein synthesis [12]. However, neither of these antimicrobials has been evaluated for their therapeutic efficacy in fish either naturally or experimentally infected with *Lactococcus* spp. in Brazil. Nevertheless, the administration of oxytetracycline in *O. mykiss* in Greece was reported to be unsatisfactory in both prophylactic and therapeutic treatments [53]. The circulation of florfenicol- and oxytetracycline-resistant strains in Brazilian fish farms could become a significant health issue when producing native species. The oral administration of amoxicillin, erythromycin, and flumequine did not yield significant results in the treatment of *O. mykiss* and *D. labrax* with lactococcosis [6,53]. However, based on the antimicrobial susceptibility tests from our study, amoxicillin and neomycin could be tested for their therapeutic efficacy against piscine lactococcosis in Brazil.

## 5. Conclusions

In this study, we calculated the pECVs for *L. garvieae* strains in Brazil. The antimicrobial resistance profiles of *L. garvieae*, *L. formosensis*, and *L. petauri* were assessed, being observed an interspecies variation. A higher percentage of resistance to various antimicrobials was observed among the evaluated isolates, especially for *L. petauri*, including multidrug-resistant strains. Resistance to florfenicol and oxytetracycline has been observed among *Lactococcus* spp. strains obtained from native fish in Brazil, which raises concerns about improper use of these drugs in the production chain of these fish species in the country. This is quite different from what has been observed in *O. niloticus* farms in Brazil, thus making it essential to monitor the susceptibility of the isolates and raise awareness among producers about the correct use of antibiotics.

## Figures and Tables

**Table 1:** Metadata of the 47 strains of lactococcosis-causing bacteria isolated from the native Brazilian fish species.

Isolate	Species	Host	Origin	Tissue	Year	State	Genbank No.	Reference
167/23-02	*L. formosensis*	*Arapaima gigas*	Farmed	Brain	2023	BA	PP591851	[2]
167/23-06	*L. formosensis*	*Arapaima gigas*	Farmed	Brain	2023	BA	PP591852	[2]
167/23-09	*L. formosensis*	*Arapaima gigas*	Farmed	Kidney	2023	BA	PQ529765	This study
AM-LG05	*L. formosensis*	*Colossoma macropomum*	Farmed	Intestine	2022	AM	PP591853	[2]
52MS	*L. formosensis*	*Pseudoplatystoma fasciatum*	Farmed	Brain	2012	MS	PP591850	[20]
LG91-23	*L. formosensis*	*Pseudoplatystoma* sp.	Farmed	Brain	2023	MG	PP591854	[2]
CRBP53	*L. garvieae*	*Arapaima gigas*	Farmed	Intestine	2023	AM	PP591857	[2]
CRBP54	*L. garvieae*	*Arapaima gigas*	Farmed	Intestine	2023	AM	PP591858	[2]
CRBP138	*L. garvieae*	*Arapaima gigas*	Farmed	Intestine	2023	AM	PP591859	[2]
CRBP144	*L. garvieae*	*Arapaima gigas*	Farmed	Intestine	2023	AM	PP591860	[2]
PA-LG01	*L. garvieae*	*Arapaima gigas*	Farmed	Brain	2018	PA	PP591868	[24]
LG88-23	*L. garvieae*	*Brycon amazonicus*	Farmed	Brain	2023	MG	PP591866	[2]
LG89-23	*L. garvieae*	*Brycon amazonicus*	Farmed	Kidney	2023	MG	PP591867	[2]
LG116-23	*L. garvieae*	*Cichla* sp.	Wild	Brain	2023	MG	PQ529771	This study
LG63-21	*L. garvieae*	*Hoplias macrophtalmus*	Farmed	Kidney	2021	MG	PP591864	[2]
LG114-23	*L. garvieae*	*Hoplias malabaricus*	Wild	Brain	2023	AM	PQ529769	This study
LG10-14	*L. garvieae*	*Lophiosilurus alexandri*	Farmed	Brain	2014	MG	PP591862	[22]
LG66-22	*L. garvieae*	*Phractocephalus hemioliopterus*	Farmed	Kidney	2022	MG	PP591865	[2]
LG09-14	*L. garvieae*	*Pseudoplatystoma corruscans*	Farmed	Kidney	2014	SP	PP591861	[22]
LG23-16	*L. garvieae*	*Pseudoplatystoma corruscans*	Farmed	Brain	2016	SP	PP591863	[23]
177	*L. garvieae*	*Pseudoplatystoma fasciatum*	Farmed	Brain	2012	MS	PP591856	[21]
31MS	*L. garvieae*	*Pseudoplatystoma fasciatum*	Farmed	Kidney	2012	MS	PP591855	[20]
LG119-24	*L. garvieae*	*Pseudoplatystoma* sp.	Farmed	Brain	2024	MG	PQ529773	This study
167/23-03	*L. petauri*	*Arapaima gigas*	Farmed	Kidney	2023	BA	PQ529760	This study
167/23-04	*L. petauri*	*Arapaima gigas*	Farmed	Kidney	2023	BA	PQ529761	This study
167/23-05	*L. petauri*	*Arapaima gigas*	Farmed	Kidney	2023	BA	PQ529762	This study
167/23-07	*L. petauri*	*Arapaima gigas*	Farmed	Kidney	2023	BA	PQ529763	This study
167/23-08	*L. petauri*	*Arapaima gigas*	Farmed	Kidney	2023	BA	PQ529764	This study
167/23-10	*L. petauri*	*Arapaima gigas*	Farmed	Spleen	2023	BA	PQ529766	This study
CRBT89	*L. petauri*	*Arapaima gigas*	Farmed	Intestine	2023	AM	PP591879	[2]
CRBT98	*L. petauri*	*Arapaima gigas*	Farmed	Intestine	2023	AM	PP591880	[2]
CRBP146	*L. petauri*	*Arapaima gigas*	Farmed	Intestine	2023	AM	PP591878	[2]
AM-LG07	*L. petauri*	*Brycon amazonicus*	Farmed	Brain	2022	AM	PP591876	[2]
AM-LG08	*L. petauri*	*Brycon amazonicus*	Farmed	Brain	2022	AM	PP591877	[2]
AM-LG02	*L. petauri*	*Colossoma macropomum*	Farmed	Intestine	2020	AM	PP591874	[2]
AM-LG03	*L. petauri*	*Colossoma macropomum*	Farmed	Intestine	2022	AM	PP591875	[2]
LG03-18	*L. petauri*	*Pseudoplatystoma corruscans*	Farmed	Brain	2018	MG	PP591881	[2]
14MS	*L. petauri*	*Pseudoplatystoma fasciatum*	Farmed	Kidney	2012	MS	PP591869	[20]
176	*L. petauri*	*Pseudoplatystoma fasciatum*	Farmed	Brain	2012	MS	PP591873	[21]
86	*L. petauri*	*Pseudoplatystoma* sp.	Farmed	Brain	2012	MS	PP591870	[21]
89/2	*L. petauri*	*Pseudoplatystoma* sp.	Farmed	Brain	2012	MS	PP591871	[21]
93	*L. petauri*	*Pseudoplatystoma* sp.	Farmed	Brain	2012	MS	PP591872	[21]
LG86-23	*L. petauri*	*Pseudoplatystoma* sp.	Farmed	Kidney	2023	MG	PP591882	[2]
LG94-23	*L. petauri*	*Pseudoplatystoma* sp.	Farmed	Brain	2023	MG	PP591883	[2]
LG104-23	*L. petauri*	*Pseudoplatystoma* sp.	Farmed	Brain	2023	MG	PP591884	[2]
LG106-23	*L. petauri*	*Pseudoplatystoma* sp.	Farmed	Kidney	2023	MG	PP591885	[2]
LG117-23	*L. petauri*	*Pseudoplatystoma* sp.	Farmed	Kidney	2023	MG	PQ529772	This study

AM: Amazonas; BA: Bahia; MS: Mato Grosso do Sul; MG: Minas Gerais; PA: Pará; SP: São Paulo.

**Table 2:** Minimum and maximum values, mean, and standard deviation of the inhibition zone diameters, epidemiological cutoff values, and wild type/non-wild type (WT/NWT) percentual for *Lactococcus* spp. and quality control strains in the antimicrobial susceptibility analysis.

Antimicrobials	Minimum Value	Maximum Value	Mean ± SD	ECV (mm)	WT (%)	NWT * (%)
*Lactococcus formosensis ^a^*						
Amoxicillin	19	27	23.2 ± 2.7	-	-	-
Erythromycin	20	30	25.7 ± 3.7	-	-	-
Florfenicol	6	28	22.3 ± 7.8	-	-	16.7
Neomycin	10	17	14.9 ± 2.4	-	-	-
Norfloxacin	6	6	6.0 ± 0.0	-	-	100
Oxytetracycline	6	27	9.5 ± 7.5	-	-	66.7
Trimethoprim-sulfametoxazole	6	6	6.0 ± 0.0	-	-	100
*Lactococcus garvieae ^b^*						
Amoxicillin	18	28	21.4 ± 2.2	≥11	100	0
Erythromycin	16	31	24.7 ± 3.7	≥16	100	0
Florfenicol	6	29	20.9 ± 4.4	≥12	94.4	5.6
Neomycin	10	19	15.1 ± 2.7	≥7	100	0
Norfloxacin	6	19	9.0 ± 4.0	-	-	47
Oxytetracycline	6	27	16.5 ± 7.3	≥10	72.2	27.8
Trimethoprim-sulfametoxazole	6	19	7.2 ± 3.4	-	-	88.2
*Lactococcus petauri ^c^*						
Amoxicillin	15	26	20.5 ± 3.0	≥16	95.8	4.2
Erythromycin	6	31	22.4 ± 7.1	≥23	66.7	33.3
Florfenicol	6	29	19.5 ± 6.9	≥21	62.5	37.5
Neomycin	10	19	14.2 ± 2.2	≥9	100	0
Norfloxacin	6	14	8.6 ± 2.9	≥13	16.7	83.3
Oxytetracycline	6	26	13.6 ± 7.5	≥23	16.7	83.3
Trimethoprim-sulfametoxazole	6	14	6.3 ± 1.4	-	-	95.8
*Escherichia coli* ATCC 25922 ^d^						
Amoxicillin	14	19	15.8 ± 2.4	-	-	-
Erythromycin	12	18	14.6 ± 2.8	-	-	-
Florfenicol	19	28	23.5 ± 4.4	-	-	-
Neomycin	16	20	18.0 ± 2.0	-	-	-
Norfloxacin	24	34	30.6 ± 5.7	-	-	-
Oxytetracycline	19	27	23.2 ± 3.3	-	-	-
Trimethoprim-sulfametoxazole	25	26	25.5 ± 0.7	-	-	-
*Aeromonas salmonicida* subsp. *salmonicida* ATCC 33658 ^d^						
Amoxicillin	24	30	27.4 ± 3.1	-	-	-
Erythromycin	19	22	20.7 ± 1.5	-	-	-
Florfenicol	32	36	34.2 ± 1.7	-	-	-
Neomycin	12	20	17.3 ± 4.6	-	-	-
Norfloxacin	21	37	29.6 ± 8.0	-	-	-
Oxytetracycline	29	32	29.7 ± 1.5	-	-	-
Trimethoprim-sulfametoxazole	24	26	25.0 ± 1.4	-	-	-

^a^ ECV undetermined; ^b^ pECV determined in this study; ^c^ pECV determined in Egger et al. [10]; ^d^ Quality control strains; * Regardless of the bacterial species, bacteria that did not present an inhibition zone and did not have a defined epidemiological cutoff value (ECV) were considered NS for the antimicrobials [31].

## Data Availability

The *gyrB* gene sequences of the *Lactococcus* spp. strains isolated from the native Brazilian fish species were included in the NCBI database as follows: *L. formosensis*—167/23-09: PQ529765; *L. garvieae*—LG114-23: PQ529769, LG116-23: PQ529771, LG119-24: PQ529773; *L. petauri*—167/23-03: PQ529760, 167/23-04: PQ529761, 167/23-05: PQ529762, 167/23-07: PQ529763, 167/23-08: PQ529764, 167/23-10: PQ529766, LG117-23: PQ529772.

## Supplementary Materials

The following supporting information can be downloaded at: https://www.mdpi.com/article/10.3390/microorganisms12112327/s1, Figure S1: Alluvial plot demonstrating the association of the host with antimicrobial susceptibility or resistance to florfenicol (FLO) and oxytetracycline (OXY) in *L. formosensis* (A), *L. garvieae* (B), and *L. petauri* (C) strains; Figure S2: Histograms of the inhibition zone for *Lactococcus garvieae* strains against amoxicillin (AMO), erythromycin (ERY), florfenicol (FLO), neomycin (NEO), norfloxacin (NOR), oxytetracycline (OXY), and trimethoprim/sulfamethoxazole (SXT); Table S1: Inhibition zones diameters (mm) of antimicrobial agents against *Lactococcus* spp. strains determined using disk diffusion susceptibility assay and MAR index calculated per isolate; Table S2: Inhibition zones diameters (mm) of antimicrobial agents against *Lactococcus garvieae* strains used to satisfy the minimum requirements of the NRI method.
