# Supplementary material for: Antimicrobial Resistance in Lactococcus spp. Isolated from Native Brazilian Fish Species: A Growing Challenge for Aquaculture
Source: Microorganisms. 2024 Nov 15;12(11):2327. doi: 10.3390/microorganisms12112327 (PMC11596430; doi:10.3390/microorganisms12112327)
Supplement: Supplementary file 1 [file microorganisms-12-02327-s001.zip › Supplementary Figure S2.pdf]

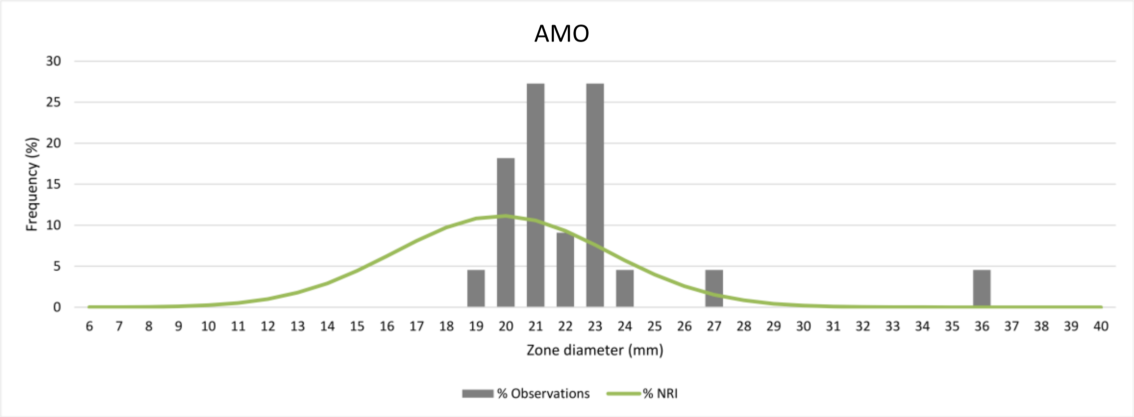
AMO
Frequency (%)
Zone diameter (mm)
% Observations
% NRI

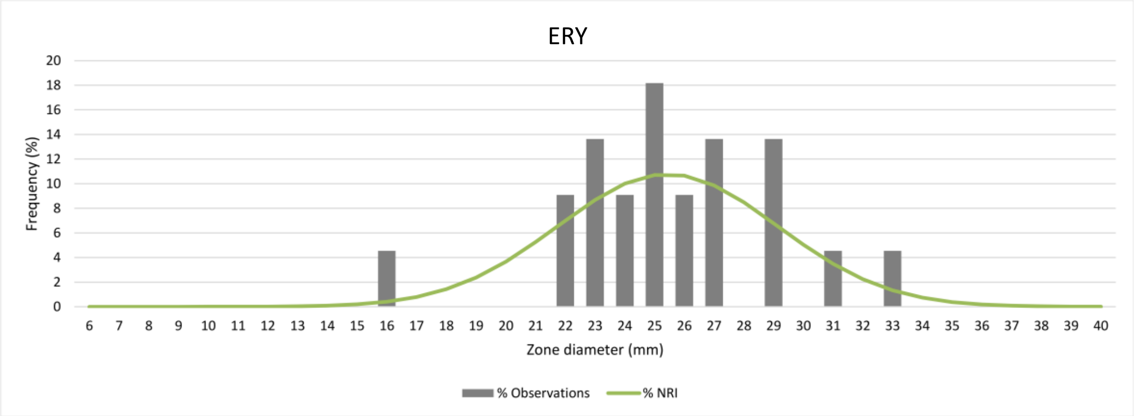
ERY
Frequency (%)
Zone diameter (mm)
% Observations
% NRI

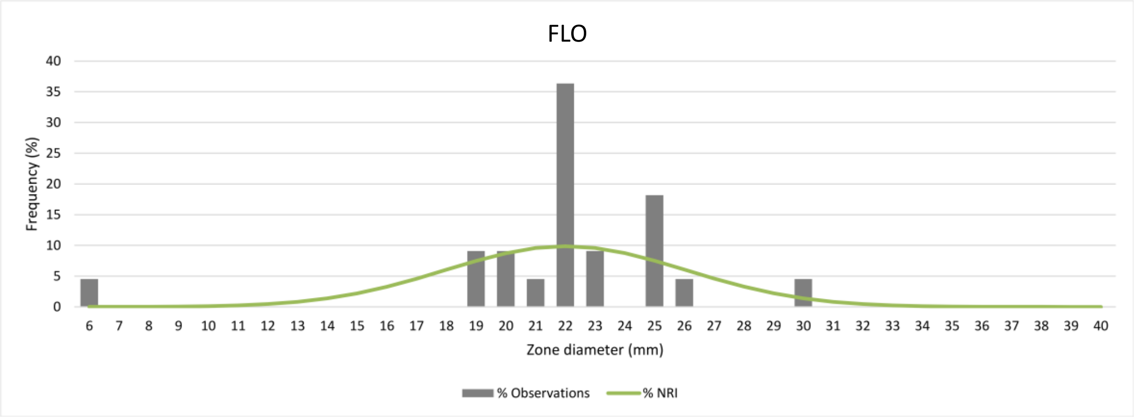
FLO
Frequency (%)
Zone diameter (mm)
% Observations
% NRI

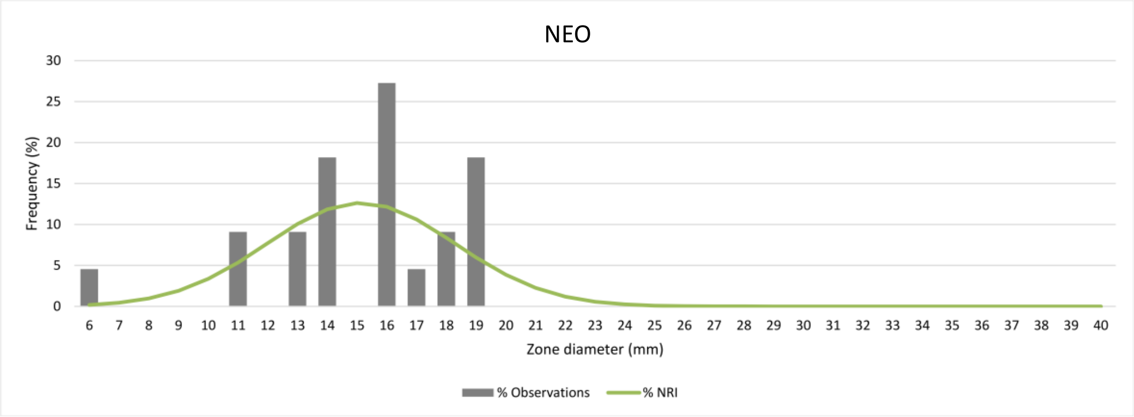
NEO
Frequency (%)
Zone diameter (mm)
% Observations
% NRI

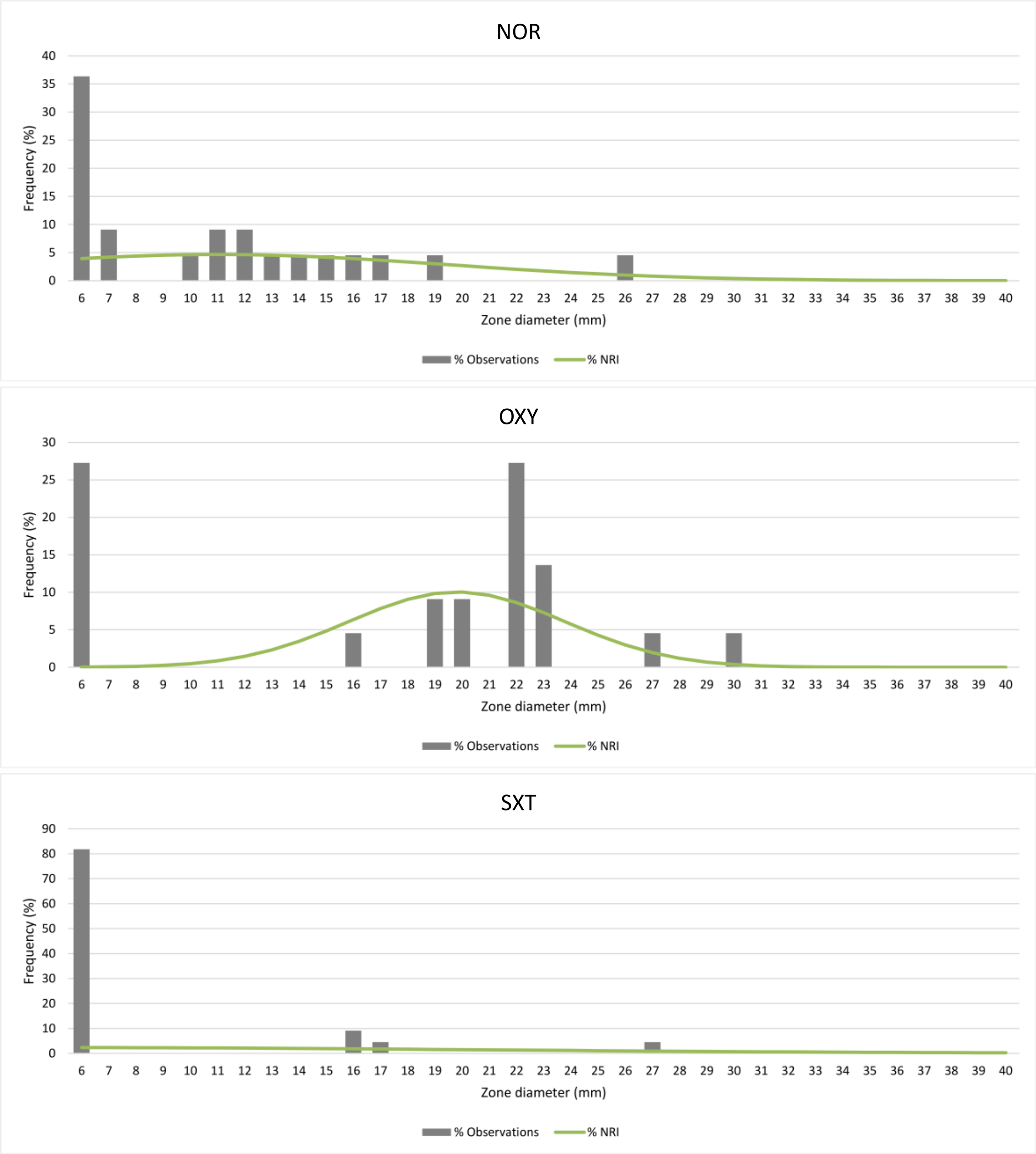

Supplementary Figure S2. Histograms of the inhibition zone for *Lactococcus garvieae* strains against amoxicillin (AMO), erythromycin (ERY), florfenicol (FLO), neomycin (NEO), norfloxacin (NOR), oxytetracycline (OXY) and trimethoprim/ sulfamethoxazole (SXT).
