# Supplementary material for: Antimicrobial Resistance in Lactococcus spp. Isolated from Native Brazilian Fish Species: A Growing Challenge for Aquaculture
Source: Microorganisms. 2024 Nov 15;12(11):2327. doi: 10.3390/microorganisms12112327 (PMC11596430; doi:10.3390/microorganisms12112327)
Supplement: Supplementary file 1 [file microorganisms-12-02327-s001.zip › Supplementary Table S1.pdf]

Supplementary Table S1. Inhibition zones diameters (mm) of antimicrobial agents against *Lactococcus* spp. strains determined using disk diffusion susceptibility assay and MAR index calculated per isolate.

Strain	Species	AMO	OXY	FLO	NOR	SXT	ERY	NEO	MARi
167/23-02*	*L. formosensis*	26	6	28	6	6	30	16	0,428571
167/23-06*	*L. formosensis*	21	6	24	6	6	23	15	0,428571
167/23-09*	*L. formosensis*	24	6	24	6	6	29	16	0,428571
52MS	*L. formosensis*	26	26	27	6	6	20	16	0,285714
AM-LG05	*L. formosensis*	25	8	27	6	6	27	10	0,285714
LG91-23*	*L. formosensis*	20	6	6	6	6	27	17	0,571429
177*	*L. garvieae*	20	6	6	6	6	16	16	0,571429
31MS	*L. garvieae*	20	20	20	6	6	24	11	0,285714
CRBP138	*L. garvieae*	21	22	22	12	6	25	14	0,142857
CRBP144	*L. garvieae*	21	22	23	6	6	29	14	0,285714
CRBP53	*L. garvieae*	21	16	20	6	6	23	13	0,285714
CRBP54	*L. garvieae*	27	27	26	7	6	31	11	0,142857
LG09-14	*L. garvieae*	21	6	19	10	6	23	17	0,285714
LG10-14	*L. garvieae*	20	19	22	6	6	25	16	0,285714
LG23-16	*L. garvieae*	23	6	22	7	6	25	19	0,285714
LG63-21*	*L. garvieae*	21	6	23	6	6	25	14	0,428571
LG66-22	*L. garvieae*	19	6	19	11	6	22	13	0,285714
LG88-23	*L. garvieae*	21	20	21	6	6	27	16	0,285714
LG89-23	*L. garvieae*	23	22	22	11	6	29	16	0,142857
LG114-23	*L. garvieae*	20	23	25	19	16	23	19	0
LG116-23	*L. garvieae*	23	22	25	16	6	29	19	0,142857
LG119-24	*L. garvieae*	23	23	22	13	16	26	18	0
PA-LG01	*L. garvieae*	22	19	22	6	6	22	14	0,285714
14MS	*L. petauri*	25	26	28	6	6	31	15	0,285714
167/23-03*	*L. petauri*	18	6	26	6	6	27	15	0,428571
167/23-04*	*L. petauri*	23	6	27	12	6	28	13	0,428571
167/23-05	*L. petauri*	26	6	24	13	6	28	12	0,285714
167/23-07*	*L. petauri*	26	6	28	10	6	28	16	0,428571
167/23-08	*L. petauri*	24	6	25	13	6	28	12	0,285714
167/23-10*	*L. petauri*	22	6	23	12	6	27	13	0,428571
176*	*L. petauri*	20	6	6	6	6	17	17	0,714286
86*	*L. petauri*	18	6	6	6	6	6	15	0,714286
89-2*	*L. petauri*	15	9	6	6	6	8	16	0,857143
93*	*L. petauri*	18	6	6	6	6	6	16	0,714286
AM-LG02*	*L. petauri*	22	20	22	6	6	23	14	0,428571
AM-LG03*	*L. petauri*	18	20	20	10	13	22	14	0,428571
AM-LG07*	*L. petauri*	19	20	19	6	6	27	16	0,571429
AM-LG08*	*L. petauri*	20	20	21	12	6	22	17	0,571429
CRBP146*	*L. petauri*	21	23	19	12	6	23	14	0,428571
CRBP89*	*L. petauri*	23	21	21	8	6	27	16	0,428571
CRBP98*	*L. petauri*	17	19	16	11	6	19	11	0,714286

LG03-18*	*L. petauri*	19	22	23	6	6	22	16	0,571429
LG86-23*	*L. petauri*	21	20	21	6	6	27	13	0,428571
LG94-23*	*L. petauri*	20	6	19	8	6	23	16	0,571429
LG104-23*	*L. petauri*	20	20	18	12	6	21	12	0,714286
LG106-23*	*L. petauri*	21	22	22	12	6	25	12	0,428571
LG117-23*	*L. petauri*	24	8	26	6	6	28	18	0,428571

Abbreviations: AMO: amoxicillin, OXY: oxytetracycline, FLO: florfenicol, NOR: norfloxacin, SXT: trimethoprim/sulfamethoxazole, ERY: erythromycin, NEO: neomycin, MARi: multiple antibiotic resistance index, * multidrug-resistant strain

Boxes in yellow show isolates classified as NWT, according to the ECV established or that did not present an inhibition zone for the antimicrobials.
