# Supplementary material for: Antimicrobial Resistance in Lactococcus spp. Isolated from Native Brazilian Fish Species: A Growing Challenge for Aquaculture
Source: Microorganisms. 2024 Nov 15;12(11):2327. doi: 10.3390/microorganisms12112327 (PMC11596430; doi:10.3390/microorganisms12112327)
Supplement: Supplementary file 1 [file microorganisms-12-02327-s001.zip › Supplementary Table S2.pdf]

Supplementary Table S2. Inhibition zones diameters (mm) of antimicrobial agents against *Lactococcus garvieae* strains used to satisfy the minimum requirements of the NRI method.

Strain	Host	AMO	OXY	FLO	NOR	SXT	ERY	NEO
A71	*Oreochromis niloticus*	23	6	25	15	6	27	16
LG02-17	*Oreochromis niloticus*	22	22	22	17	6	27	18
LG13-19	*Oreochromis niloticus*	36	30	30	26	27	33	6
LG64-21	*Xiphophorus maculatus*	23	22	22	12	6	24	16
LG115-23	*Trichogaster lalius*	24	23	25	14	17	26	19

Abbreviations: AMO: amoxicillin, OXY: oxytetracycline, FLO: florfenicol, NOR: norfloxacin, SXT: trimethoprim/sulfamethoxazole, ERY: erythromycin, NEO: neomycin
