# Supplementary figures and images for: Antimicrobial Resistance in Lactococcus spp. Isolated from Native Brazilian Fish Species: A Growing Challenge for Aquaculture
Source: Microorganisms. 2024 Nov 15;12(11):2327. doi: 10.3390/microorganisms12112327 (PMC11596430; doi:10.3390/microorganisms12112327)

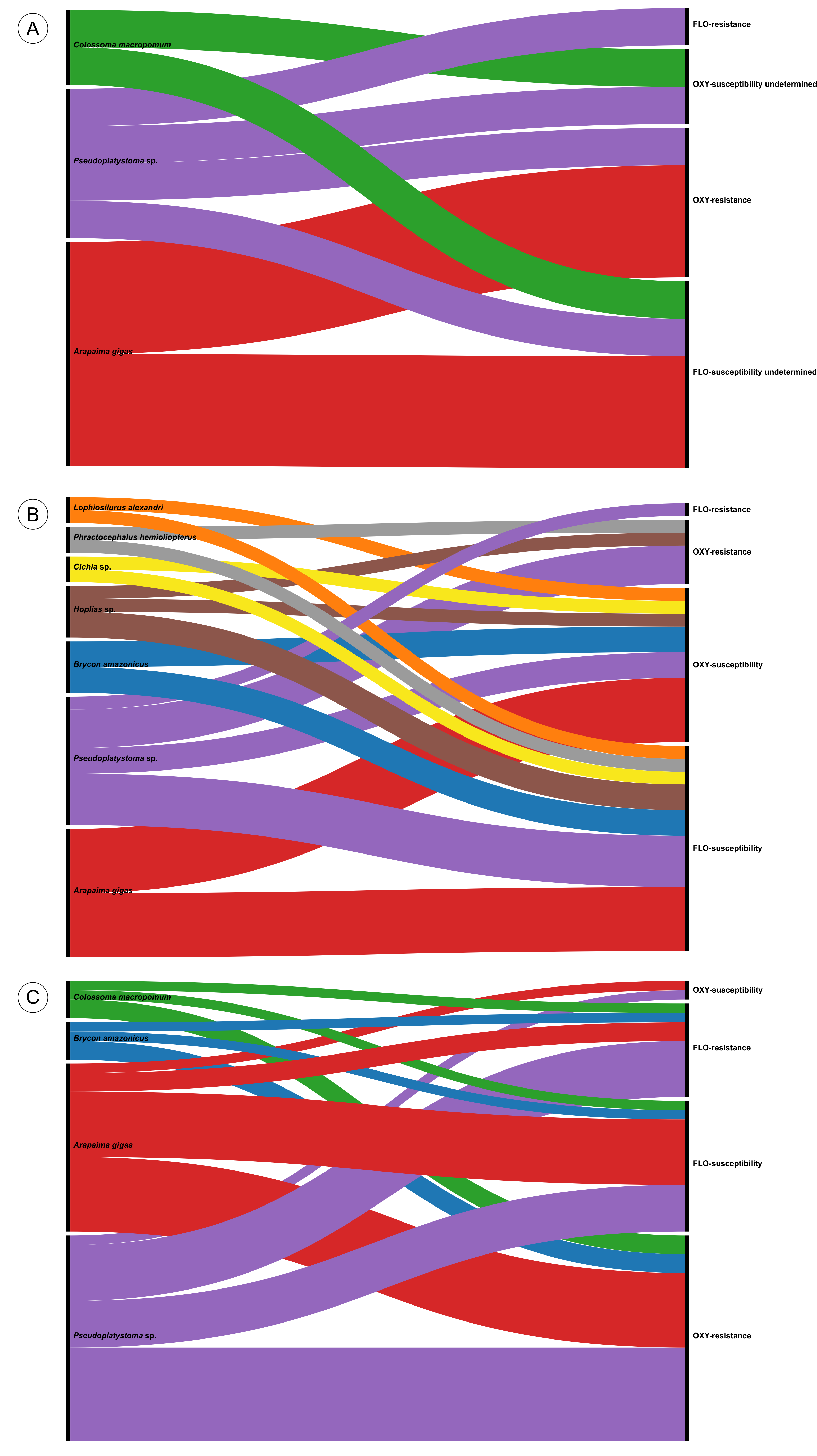

Supplement: Supplementary file 1 [file microorganisms-12-02327-s001.zip › Supplementary Figure S1.png]
